# Anastomotic stricture prediction in patients with esophageal atresia with distal fistula

**DOI:** 10.1007/s00383-023-05423-z

**Published:** 2023-02-22

**Authors:** Natalia Newland, Jiri Snajdauf, Alena Kokesova, Jitka Styblova, Ondrej Hradsky, Isabel Meusel, Barbora Kucerova, Martin Kyncl, Magdalena Simsova, Vladimir Mixa, Michal Rygl

**Affiliations:** 1 Department of Pediatric Surgery, 2nd Faculty of Medicine, Charles University in Prague and Motol University Hospital, Prague, Czech Republic; 2 Department of Pediatric Gastroenterology, 2nd Faculty of Medicine, Charles University in Prague and Motol University Hospital, Prague, Czech Republic; 3 Second Faculty of Medicine, Charles University in Prague and Motol University Hospital, Prague, Czech Republic; 4 Department of Radiology, 2nd Faculty of Medicine, Charles University in Prague and Motol University Hospital, Prague, Czech Republic; 5 Department of Anesthesiology and ICM, 2nd Faculty of Medicine, Charles University in Prague and Motol University Hospital, Prague, Czech Republic

**Keywords:** Esophageal atresia, Stricture, Risk factors, Esophagram, Stricture index

## Abstract

**Purpose:**

To investigate potential early risk factors for anastomotic stricture formation and assess the predictive role of post-operative esophagrams.

**Methods:**

A retrospective study of patients with esophageal atresia with distal fistula (EA/TEF) operated between 2011 and 2020. Fourteen predictive factors were tested for stricture development. Esophagrams were used to calculate early (SI1) and late (SI2) stricture index (SI = anastomosis diameter/upper pouch diameter).

**Results:**

Of 185 patients operated for EA/TEF in the 10-year period, 169 patients met the inclusion criteria. Primary anastomosis was performed in 130 patients and delayed anastomosis in 39 patients. Stricture formed in 55 patients (33%) within 1 year from anastomosis. Four risk factors showed strong association with stricture formation in unadjusted models: long gap (*p* = 0.007), delayed anastomosis (*p* = 0.042), SI1 (*p* = 0.013) and SI2 (*p* < 0.001). A multivariate analysis showed SI1 as significantly predictive of stricture formation (*p* = 0.035). Cut-off values using a receiver operating characteristic (ROC) curve were 0.275 for SI1 and 0.390 for SI2. The area under the ROC curve demonstrated increasing predictiveness from SI1 (AUC 0.641) to SI2 (AUC 0.877).

**Conclusions:**

This study identified an association between long gap and delayed anastomosis with stricture formation. Early and late stricture indices were predictive of stricture formation.

## Introduction

Since the first successful repair of esophageal atresia (EA) by Cameron Haight in 1941, the focus of today´s management is evolving toward improving the quality of peri-operative and post-operative care and addressing associated morbidity in order to improve quality of life [1, 2].

Anastomotic stricture has an incidence reported between 32 and 80% [3–6], and remains the most common complication of EA repair. It represents a demanding and time-consuming burden for both families and health care providers. One of the challenging aspects in stricture management is the identification of patients at high risk for stricture development and detecting predictive signs, especially in the early post-operative period before the clinical symptoms occur. Such prediction could potentially facilitate better tailored care for these patients who are at an increased risk of emergency complications of anastomotic strictures such as food impactions or aspiration and also slowly presenting complications resulting from long-standing dysphagia, such as malnutrition leading generally to a poorer quality of life [7].

The objectives of this study are first, to assess the association strength of well-established factors with stricture formation. Second, we aim to test the predictive capacity of post-operative esophagrams in stricture development, focusing particularly on the early esophagram and the usefulness of two consecutive esophagrams. Third, we aim to identify the optimal combination of factors that might give an early indication of a high risk of stricture development.

## Materials and methods

### Study population

**Fig. 1:**
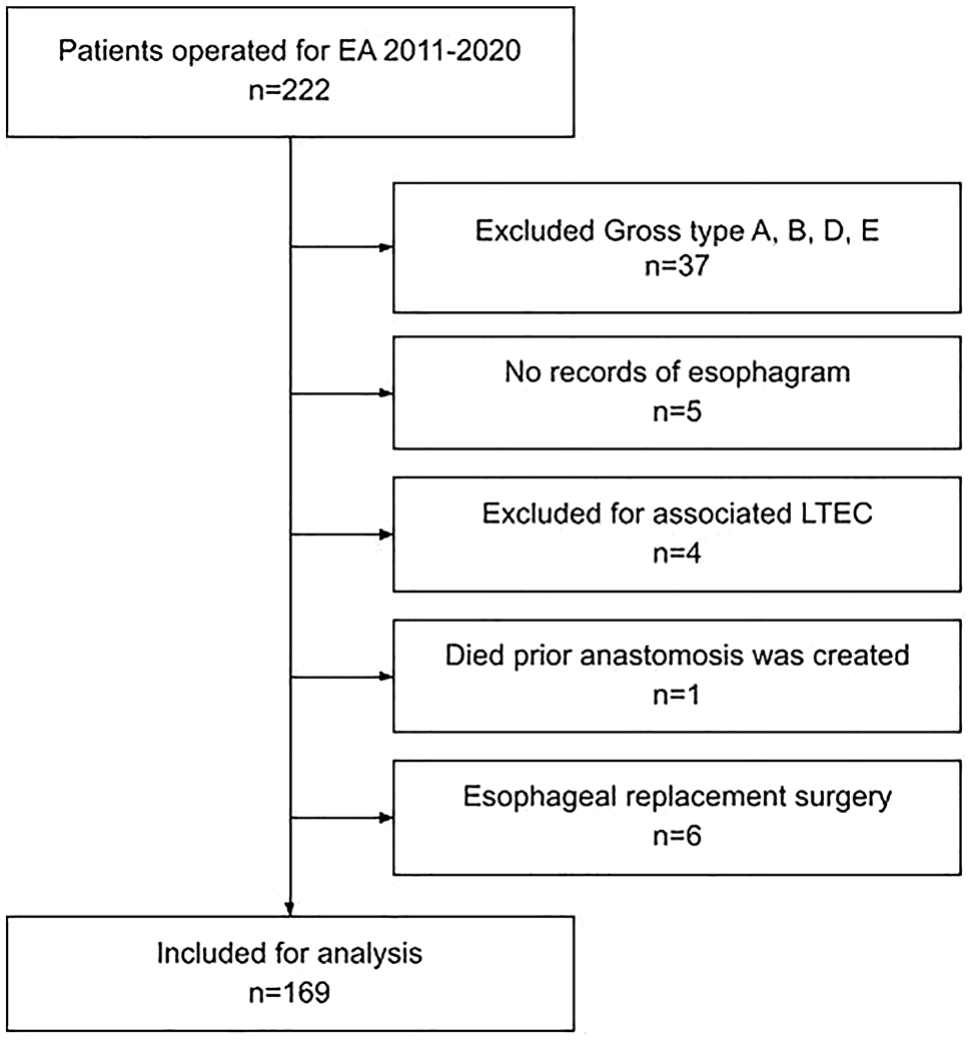
Flowchart of patient inclusion and exclusion criteria

Following institutional ethical committee approval, a retrospective longitudinal cohort study of all patients with EA treated at our Pediatric Surgery Department was performed. Electronic records of patients operated in a 10-year period between 1 January 2011 and 31 December 2020 were reviewed. To provide a more homogenous population for investigation of factors affecting stricture formation, only patients with distal fistula were included for the analysis. Further requirements for inclusion were that patients underwent a primary or delayed anastomosis, had two consecutive esophagrams and had at least 1 year of follow-up. Patients with associated laryngotracheoesophageal cleft (LTEC) were excluded from the analysis (Fig. 1). Comparative analysis was performed between groups with and without anastomotic strictures.

The extracted data included 14 factors which were tested for association with stricture formation and its strength. Those included gender, gestational age, birth weight, associated cardiac anomalies, the type of anastomosis (primary, delayed), long gap, length of post-operative ventilation, anastomotic leak on early post-operative esophagram, recurrent fistula, two stricture indices, use of anti-reflux medication (at our institution, proton-pump inhibitors) and anti-reflux surgery (Nissen or Boix-Ochoa fundoplication) (Table 1). As the focus of the study is on detecting predictive factors before the stricture forms, the data do not include the clinical symptoms of anastomotic stricture.

**Table 1:** 14 factors tested for association with stricture formation

Variable	Stricture (*n* = 55)	No Stricture (*n* = 114)	OR, 95%CI	*P* value
Male	32 (58.2%)	61 (53.5%)	1.20; 0.63–2.33	0.56
Birthweight, kg Median (Q1, Q3)	2.35 (2.02–3.06)	2.61 (1.78–3.06)	1; 0.99–1	0.32
Gestational age, wk Median (Q1, Q3)	36 (34, 38)	38 (35, 39)	0.94; 0.85–1.04	0.25
Associated cardiac anomaly	13 (23.6%)	23 (20.2%)	1.22; 0.55–2.62	0.60
Long gap EA	17 (30.9%)	15 (13.2%)	2.95; 1.34–6.57	0.007
Primary anastomosis	37 (67.3%)	93 (81.6%)	0.46; 0.22–0.97	0.04
TSK surgery	0 (0%)	2 (1.8%)	0	0.20
Length of ventilation, days Median (Q1, Q3)	6 (4, 9)	4 (3, 8)	1; 0.98–1.02	0.91
SI1 Median (Q1, Q3)	0.33 (0.25, 0.43)	0.43 (0.33, 0.56)	0.06; 0.006–0.56	0.01
SI2 Median (Q1, Q3)	0.22 (0.15, 0.36)	0.55 (0.43, 0.67)	0	0
Leak	14 (25.5%)	15 (13.2%)	2.25; 0.99–5.11	0.05
Recurrent fistula	6 (10.9%)	4 (3.5%)	3.36; 0.92–13.67	0.06
PPI treatment	45 (81.8%)	69 (60.5%)	2.93; 1.39–5.47	0.003
Fundoplication performed	2 (3.6%)	5 (4.4%)	1.04; 0.34–2.84	0.94

Anastomotic stricture was defined according to Krishanan et al. as a narrowing at the site of anastomosis seen on esophagram accompanied by clinical symptoms requiring at least one dilatation [1]. Long gap was defined as a distance between upper and lower pouches not allowing for primary anastomosis, as per the decision of a consultant surgeon.

### Esophagrams

**Fig. 2:**
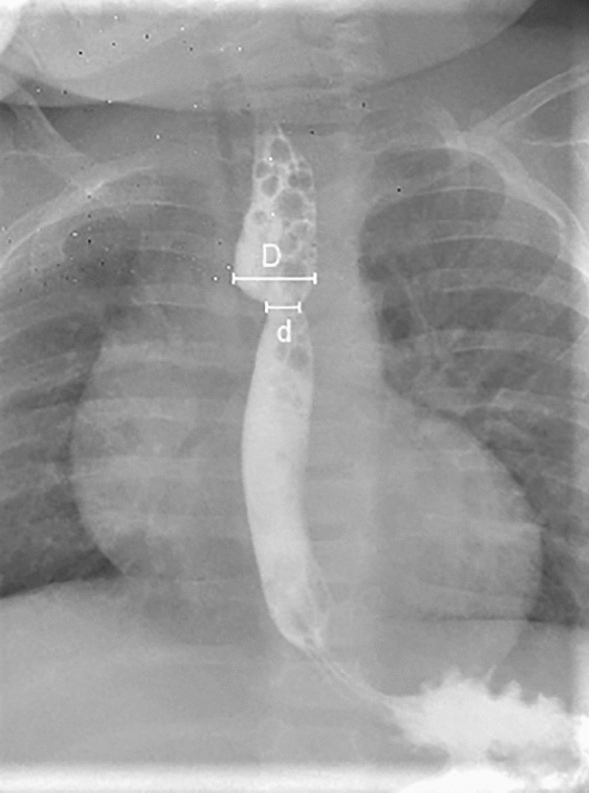
Calculation of stricture index (SI) by dividing the minimum anastomotic diameter (d) by the maximum upper pouch diameter (D) in antero-posterior view in millimetres, SI = d/D

Esophagrams were performed after the 7^th^ day post-operatively to rule out anastomotic leak prior to the initiation of oral feeds. This was defined for the purpose of this study as an “early” esophagram. A second, “late” esophagram was performed at a routine follow-up 2 months from anastomosis creation. Both esophagrams were used to calculate stricture indices (SIs) by dividing the minimum anastomotic diameter (d) by the maximum upper pouch diameter (D) in anterior–posterior view in millimetres, SI = d/D (Fig. 2). SI1 was calculated from the early and SI2 from the late esophagrams. All SIs were measured by a single physician, who was not blinded to other clinical information. Values were compared for patients with and without stricture. The lower the SI, the more severe the stricture.

### Statistical analysis

All data were analyzed using R statistical software (version 4.2.0). Continuous variables were described as median and interquartile range (IQR). Categorical variables were described as absolute frequencies and percentages. The association of predictors with stricture development was tested using a univariate generalized linear regression model. To identify early predictors of stricture development, a multivariate generalized linear regression model was constructed. Receiver operating characteristic (ROC) curves and the area under the ROC curve (AUC) were calculated using R package “pROC”. For the purpose of estimating the model accuracy based on available predictors (with calculated cut-offs), we constructed a multivariate generalized linear regression model and a random forest model using R package “caret”.

## Results

Over the 10-year period between 1 January 2011 and 31 December 2020, a total of 222 patients with esophageal atresia were treated at our tertiary centre. Of those, 185 patients had esophageal atresia with distal fistula (EA/TEF). The inclusion criteria were met by 169 patients. The reasons for exclusion were: four associated LTECs, six gastric replacement surgeries, five patients had no records of the early esophagram and one patient died before an anastomosis was created (Fig. 1).

Our study group consisted of 93 girls and 76 boys, with a median birth weight of 2580 g (1910–3080 g) and a median gestational age of 37.0 weeks (34–39 weeks). Of the 169 patients, 55 developed an anastomotic stricture in the first year of life, which represents 33% of the study group. The median time to stricture formation was 78 days (61–120 days) and the median number of dilatations required in the first year of life was 2 (1– 4).

Patients with and without stricture were comparable in terms of demographic data: gender, birth weight and gestational age. Although patients with stricture were slightly younger with a smaller birth weight, these data were not significantly associated with stricture development. Cardiac anomaly was found in 36 patients (21.3%) and of them 13 developed a stricture (36%), compared with 32% of patients who did not have a cardiac anomaly and developed a stricture (OR = 1.22, 95%CI 0.55–2.62, *p* = 0.609).

All but two patients underwent an open repair using a right posterolateral thoracotomy via the fourth intercostal space. Primary anastomosis was performed in 130 patients (77%) and stricture formed in 37 patients of those (28.5%). Delayed anastomosis was performed in 39 patients (20%) and stricture developed in 46.2% of them. Upon univariate analysis, delayed anastomosis was associated with stricture formation more strongly than primary anastomosis (OR = 0.46, 95%CI 0.22–0.97, *p* = 0.042).

**Fig. 3:**
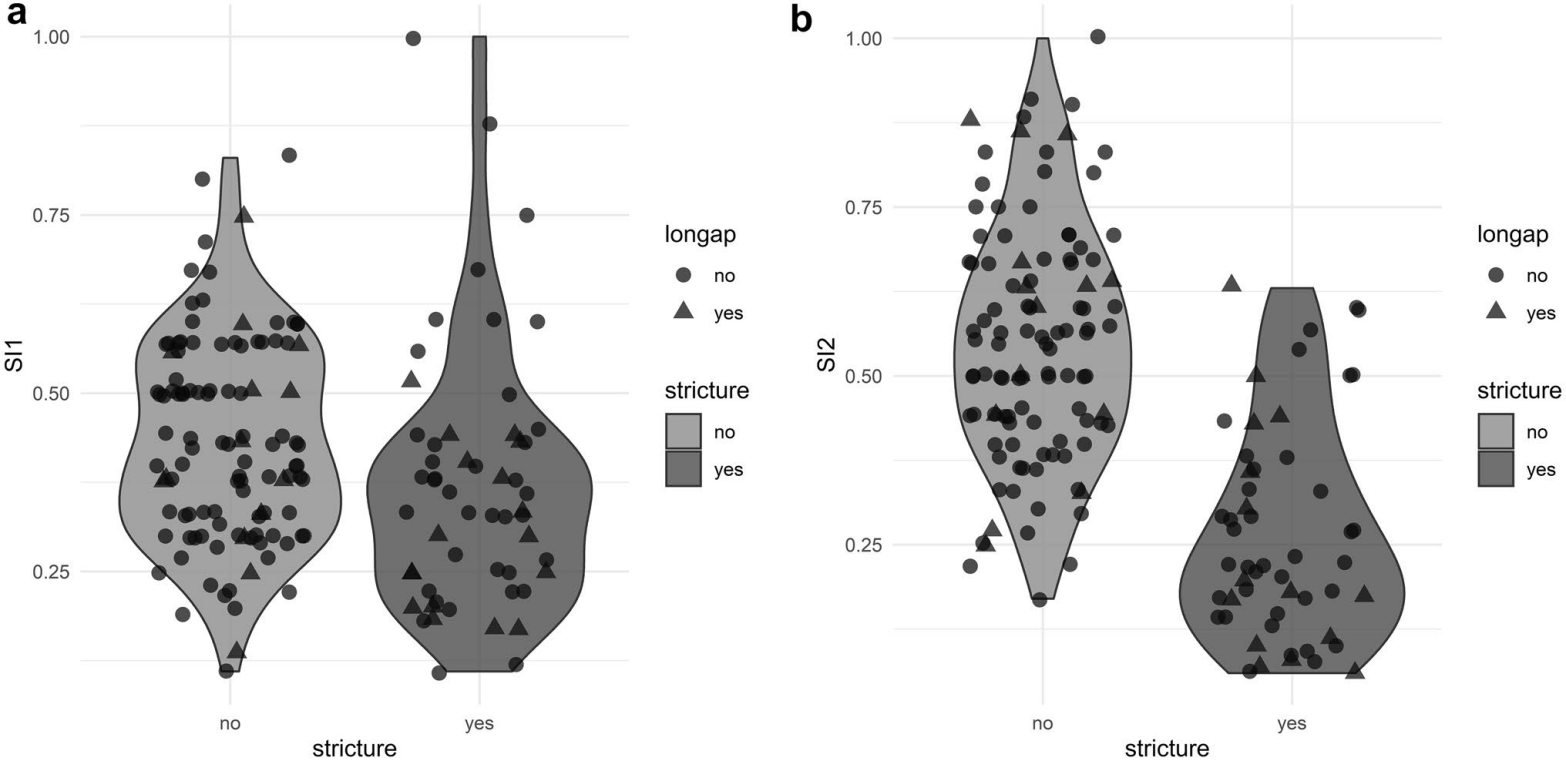
**a, b** The range of values for SI1 and SI2 in patients with and without strictures

**Fig. 4:**
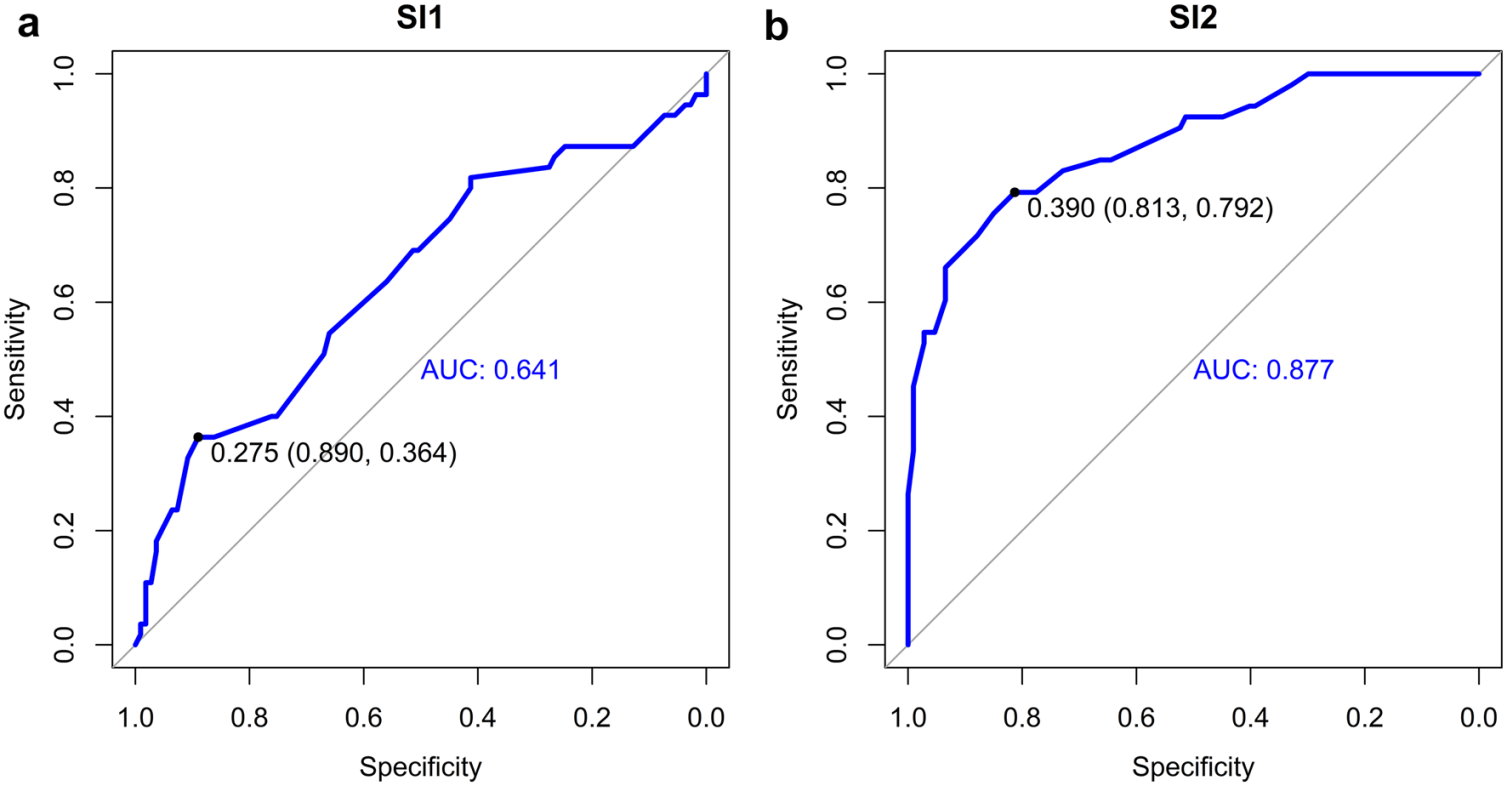
**a, b** Receiver operating characteristic analysis showing the cut-off ratios for SI1 and SI2 and demonstrating an increasing predictability from the early to the late esophagram

Apart from delayed anastomosis, we found three other risk factors that were strongly associated with stricture formation: long gap (*p* = 0.007), SI1 (*p* = 0.013) and SI2 (*p* < 0.001), Table 1. Long gap was found in 32 patients of the total (19%). Of those, 53% developed a stricture in comparison with 28% of patients who did not have a long gap EA but developed a stricture (OR = 2.95, 95%CI 1.34–6.57, *p* = 0.007). Both SI1 and SI2 were statistically significantly associated with stricture formation (OR = 0.06, 95%CI 0.00–0.56, *p* = 0.013 and OR = 0, 95%CI 0–0.001, *p* < 0.001, respectively). The median values for SI1 were 0.330 (0.250—0.435) for patients who developed a stricture and 0.430 (0.330—0.560) for patients with no stricture. The median values for SI2 were 0.220 (0.150—0.360) for patients with stricture and 0.550 (0.430—0.670) where no stricture developed. The range of values for SI1 and SI2 is demonstrated in Fig. 3a, b. On a ROC analysis, the best cut-off ratios were 0.275 for SI1 and 0.390 for SI2 with the AUC showing 0.641 for SI1 (sensitivity 86%, specificity 36%) and 0.877 for SI2 (sensitivity 81%, specificity 79%), Fig. 4a, b.

The following three factors did not show an association with future stricture formation in our study. Anastomotic leak identified on an early post-operative esophagram was found in 29 patients (17%). Leak management included conservative treatment in 23 patients, re-anastomosis in 5 patients and chest drain placement in 1 patient. Patients with a leak were at an increased risk for stricture formation (25.5%) compared with patients who had no leak and developed a stricture (13%), however, this finding was not statistically significant (OR = 2.25, 95%CI 0.99–5.11, *p* = 0.052). Recurrent fistula was only present in ten of the EA/TEF patients (6%) and of those, six developed a stricture (60%, OR = 3.36, 95%CI 0.92–13.67, *p* = 0.066). The median length of post-operative ventilation was 5 days (3–8 days) and had no significant effect on stricture formation (OR = 1, 95%CI 0.98–1.02, *p* = 0.912).

The last two tested factors were PPI treatment and fundoplication. Eighty-nine patients (53%) in our cohort had symptoms of GERD and were, therefore, prescribed PPIs. Of those, 43% developed a stricture despite being on the anti-reflux medication. Of the 79 patients (47%) who had no clinical symptoms of GERD and hence were not given PPIs, 22% developed a stricture. A univariate analysis showed that patients taking PPIs developed stricture at a statistically significantly higher rate (OR = 2.93, 95%CI 1.38–6.70, *p* = 0.004). Fundoplication was only performed in seven patients (4%) within the first year of follow-up and two of them developed a stricture which did not prove to be associative.

Lastly, all mentioned factors were analyzed in multivariate generalized linear regression models. In the first analysis, we tested which factors could be responsible for stricture formation. The final model included long gap, presence of recurrent fistula and PPI treatment (patients with clinical symptoms of GERD). Long gap and PPI both showed independent associations with stricture formation (OR = 2.56, 95%CI 1.11–5.98, *p* = 0.02 and OR = 2.24, 95%CI 1.11–4.63, *p* = 0.02, respectively). The second multivariate analysis was performed to find out which of the factors could help predict a future stricture formation. This model included SI1, long gap, recurrent fistula, thoracoscopic surgery and PPI treatment, and we found that both SI1 and PPI treatment were predictors of an increased risk for stricture formation (OR = 0.07, 95%CI 0.00–0.84, *p* = 0.035 and 0R = 2.32, 95%CI 1.13–4.86, *p* = 0.021, respectively). Lastly, using the random forest method with all predictors, we constructed a model with an accuracy of 68% when a cut-off value of < 0.275 for SI1 was applied.

## Discussion

This study provides a thorough examination of multiple risk factors associated with stricture formation and investigates the predictive capacity of post-operative esophagrams. In addition to examining the well-known factors, the main focus was in evaluation of the strength of the early signs in detecting patients at higher risk of future stricture development, specifically before the symptoms occur. Such prediction could help prevent the emergency and long-standing complications of anastomotic strictures. Stricture rate in our cohort, 55 patients of 169 (33%), was comparable with or lower than rates reported in other recently published single centre studies [4, 8–10]

The primary interest of this study was in identifying the potential role of early post-operative esophagram in stricture prediction. The post-operative esophagram as a routine diagnostic procedure prior to initiation of feeds is performed by 72% and 85% of pediatric surgeons according to the two surveys from the past decade [11, 12] and our question was whether additional information could be obtained from this diagnostic procedure. Some studies contest the usefulness of contrast studies in early management for the purpose of leak detection [13, 14] and stricture prediction [15]. However, we argue that an objective measure early in a patient’s management could provide an important initial awareness, help identify patients at higher risk, and facilitate more tailored care and better parent counseling.

Several authors have previously proposed the use of stricture index measured on contrast esophagrams to predict the severity of strictures. The first such index was published by Said et al. who compared the widest diameter of the lower pouch and the stricture diameter [16]. Since then, more complex indices have been examined, such as upper pouch esophageal anastomotic stricture index (U-EASI), lower pouch esophageal anastomotic stricture index (L-EASI), lateral SI (L-SI), and anterior/posterior SI (AP-SI), with varying predictive value [16–18]. Authors often debate whether upper or lower pouch to stricture diameter ratio is a more accurate measure.

We chose a stricture index measured as a ratio between the anastomotic diameter and the upper pouch diameter, given that the majority of symptoms which drive the need for esophageal dilatation are caused by food pooling in the dilated upper pouch. Further, the two dimensions typically represent the extreme widths of the esophagus and, therefore, highlight the critical anatomical predispositions. Our data show that the early stricture index (SI1) is a predictor of stricture development in the first year of life, with AUC 0.641 and the cut-off value of 0.275 on a ROC curve. Further, using the cut-off value for SI1 with a combination of other factors (long gap, recurrent fistula, thoracoscopic surgery and PPI treatment), we created a predictive model and tested its potential for stricture prediction. The accuracy of this model was 68% and SI1 and PPI treatment (patients with clinical symptoms with GERD) emerged as the most significant risk factors. Therefore, we propose that in addition to the utilization of the early post-operative esophagram in ruling out a leak prior to initiation of oral feeds, an early stricture prediction could also be surmised from this diagnostic procedure.

Amongst the publications on stricture index utilization, Landisch et al. presented results of the U-EASI and also showed a correlation between the ratio of the upper pouch and anastomosis diameter and stricture development. Interestingly, although they used a more comprehensive index by also including a lateral view, their cut-off ratios for SI1 and SI2 were very similar to ours. Further, they also suggest that U-EASI is relevant in reflecting symptoms of stricture [18]. Similarly, a publication by Ordóñez et al. also supports upper pouch stricture ratio as a useful tool for stricture prediction [8]. Lastly, a recent study by Huang et al. compared various indices to find the upper pouch stricture index to be superior [19]. Another interesting proposal for stricture measurement was published by Parolini et al. in 2013 where stricture index was measured at a routine endoscopy 1 month after surgery rather than on an esophagram, however, this method requires an additional general anesthesia. They also utilized a ratio of upper pouch to stricture diameter and found a statistical significance between the stricture index and number of dilatations required [20]. In contrast, in a study published by Sun et al., lower pouch esophageal anastomotic stricture index (L-EASI) showed superiority in stricture prediction compared with U-EASI. They propose that L-EASI is a more accurate measure of the true esophageal diameter [17].

Another point of consideration in utilizing stricture indices is the potentially higher predictiveness of the late esophagram over the early post-operative one. The present study shows that the second esophagram correlates more closely with stricture formation than the first one (AUC 0.877 and 0.641, respectively) with a cut-off value of 0.390 on a ROC curve. A similar experience was published in three other papers where indices calculated from early esophagrams did not show significant association with dilatation in comparison with the late esophagrams [8, 18, 19]. This result is not surprising and reflects the clinical nature of the stricture development, where the initial subtle symptoms in a neonate become clinically apparent in an infant on full oral feeds at a later follow-up. Even though late esophagrams provide more certainty, they also impose an extra radiation exposure which has to be considered. On the other hand, a number of patients with anastomotic stricture present with emergency complications, such as food impactions and aspiration and according to some studies aspirations may contribute to a significant number of late deaths in the first year of life [21, 22]. Therefore, a balance between the benefits of the additional information and risks of the radiation exposure needs to be considered. All in all, our data suggest that the early esophagram may be a sufficient alert to exercise vigilance and could serve in selecting patients for high-risk management.

Two other tested factors in our study, delayed anastomosis and long gap EA, were strongly associated with stricture formation and both also provided early information. Delayed anastomosis and long gap partly overlap each other. While all our patients with long gap underwent a staged procedure, patients with lower birth weight, an unstable condition or with severe associated anomalies would in many cases also undergo a ligation of the TEF in the first stage and an anastomosis in the second stage. Both factors showed a significant association in univariate analysis, however, the multivariate analysis highlighted that long gap plays a role as an independent significant risk factor responsible for stricture formation. The significance of both factors in stricture formation is supported in numerous other publications [5, 23–25] and the associations correspond with the fact that both predispose for higher tension of the anastomosis and trickier repair [9].

Leak on post-operative esophagram is often described as one of the contributing factors for stricture formation and also could serve as an early indicator. In our study, 25.5% of patients with leak developed a stricture, in comparison with 13% of patients who had no leak and developed a stricture. Even though our data demonstrated an increased risk for stricture formation in patients with leak, we did not prove a statistically significant association, contrary to previously published studies [9, 10], however, this is likely due to a small number of patients with leak in our cohort. Demographic data, including gender, birth weight and gestational age did not show any significant association in our study, which is in agreement with publications by other authors [20, 26].

GERD is a suspected contributor for stricture formation with a published prevalence of 20–63% among patients with EA [6]. Current recommendations by ESPGHAN-NASPGHAN suggest treating all EA patients with acid suppression within the first year of life [1]. Despite previous publications stating that clinical symptoms were not a good predictor of GERD in infants [27], PPI treatment at our centre is indicated individually, based on GERD symptom recognition, such as frequent regurgitation, coughing, aspiration episodes or Sandifer syndrome. The reason for selective PPI treatment at our institution is the growing evidence of potential side effects of PPI treatment, such as increased risk for gastrointestinal and respiratory infections, vitamin B12 deficiency, osteoporosis, changes in intestinal microflora and an increased risk of necrotising enterocolitis in premature infants [28–31]. Our data show that patients who were indicated for anti-reflux treatment because of their clinical manifestation of GERD were at higher risk for stricture formation despite taking PPIs compared with patients who had no clinical presentation of GERD and were, therefore, not prescribed PPIs. Further, PPI treatment showed a significant independent statistical association with stricture formation on both multivariate models. Assuming our diagnosis of GERD by symptom recognition was accurate, our data suggest that PPIs did not stop the stricture from forming, as has been previously published by several other authors [4, 18, 32–35]. Fundoplication was also examined for association with stricture formation, however, the number of patients who underwent anti-reflux surgery was too small to make any conclusions. Therefore, we found no association between fundoplication and stricture formation, which reflects previous studies [9]. Overall, the clinical suspicion of GERD could also include other risk factors not related to GERD which could be the true risk factors for stricture development and thus were not modified by PPI treatment or fundoplication.

One of the strengths of the current study is the larger sample size compared with the published literature to date. Another strong feature is the homogeneity of the patient group, resulting from a standardized treatment handled in a single institution, where all surgeries and post-operative care were led by one team of specialists dedicated to patients with EA. However, the retrospective nature of this study is a limitation and involved a small data loss. Other limitations include the subjective classification of long gap EA as well as the symptom-based diagnosis of GERD.

## Conclusion

This study proposes that identifying risk factors and utilizing simple and easy to interpret predictive measures should become a part of routine screening for anastomotic stricture in early management of patients with EA before clinical symptoms arise. We found that of the possible risk factors responsible for stricture formation, long gap, delayed anastomosis, SI1 and SI2 showed significant associations in a univariate analysis. As independent factors, both long gap and SI1 demonstrated good predictive values. Even though SI2 was also identified as predictive of stricture formation, we believe that this diagnostic procedure should only be considered in selected patients who are at high-risk in order to avoid additional radiation exposure. This study further suggests that a model incorporating early SI in addition to the other risk factors, could help create a tool for stricture prediction and this should ideally be tested in a prospective multi-centre study.

## Data Availability

The datasets generated during and/or analysed during the current study are available from the corresponding author on reasonable request.
